# Risk factors associated with IgG seropersistence to *Chlamydia trachomatis* and *Mycoplasma genitalium*

**DOI:** 10.1017/S095026882500007X

**Published:** 2025-01-24

**Authors:** Nea Koskela, Julia Butt, Birgitta E. Michels, Kari Syrjänen, Seija Grenman, Tim Waterboer, Stina Syrjänen, Karolina Louvanto

**Affiliations:** 1 Department of Obstetrics and Gynecology, Faculty of Medicine and Health Technology, Tampere University, Tampere, Finland; 2 German Cancer Research Center (DKFZ), Heidelberg, Germany; 3 SMW Consultants, Ltd., Kaarina, Finland; 4 Department of Obstetrics and Gynecology, Turku University Hospital, University of Turku, Turku, Finland; 5 Department of Oral Pathology, Institute of Dentistry, Faculty of Medicine, University of Turku, Turku, Finland; 6 Department of Pathology, Turku University Hospital, Turku, Finland; 7 Department of Obstetrics and Gynecology, Tampere University Hospital, Tampere, Finland

**Keywords:** Bacterial infections, Chlamydia, Mycoplasma, Serology, Sexually transmitted infections (STIs)

## Abstract

Sexually transmitted infections caused by *Chlamydia trachomatis (Ct)* and *Mycoplasma genitalium (Mg)* have significant implications both at the individual and societal levels. Our study evaluated various co-factors associated with persistent serum IgG-antibodies to *Ct* and *Mg.* Three hundred and twenty nine pregnant women and 135 men from the Finnish Family HPV study were analyzed for serum IgG-antibodies of pGP3 for *Ct* and MgPa and rMgPa for *Mg* using multiplex serology. Seropersistence to both *Ct* and *Mg* was more common in women (30.4% and 13.3%) than in men (17.4% and 5.3%). The number of lifetime sexual partners above 10, the practice of anal sex, and a history of diagnosed *Ct* were associated with seropersistence to *Ct* in women, adjusted ORs 5.6 (95%CI 1.39–22.29), 15.3 (95%CI 1.18–197.12) and 18.0 (95%CI 5.59–57.92), respectively. The increasing number of partners before the age of 20 was the main risk factor for seropersistence among women with *Mg*, adjusted OR range from 5.0 to 12.3 (95%CI range 1.17–100.90) and in men only with 6–10 partners for *Ct*, adjusted OR 12.6 (95%CI 1.55–102.49). To conclude, persistent *Ct* antibodies were associated with various sexual activities, and *Mg* seropositivity was mainly associated with increased sexual activity in early adulthood.

## Introduction

The outcome of sexually transmitted *Chlamydia trachomatis* (*Ct*) and *Mycoplasma genitalium* (*Mg*) infections can be severe because of their potentially serious sequels on reproductive health, including pelvic inflammatory disease (PID), salpingitis, and tubal infertility [1,2]. Globally, infections caused by *Ct* are the most prevalent sexually transmitted infections (STIs), second only to the parasite *Trichomonas vaginalis* (*Tv*) infections [2]. According to global estimates, there are nearly 129 million new cases of *Ct* infections worldwide each year [3]. To date, comprehensive global studies assessing the prevalence of *Mg* remain absent. However, a recent meta-analysis concluded the prevalence of *Mg* to be 1.3% and 3.9% in developed and developing countries, respectively [4]. Importantly, both pathogens can cause asymptomatic infections, which are challenging in terms of diagnosis and treatment.

*Ct* and *Mg* present with analogous clinical behavior, causing similar clinical manifestations or being asymptomatic [1,2,5,6]. Case–control studies have shown that clinical carriage of *Ct* and *Mg* can be independent of one another [6,7]; however, a study of female sex workers showed women with *Ct* and *N. gonorrhea* infection had an increased risk of *Mg* [8]. This correlation is understandable as they share the same route of transmission. Additionally, a recent study at the National Sexual Health Clinic in Singapore found that *Mg* was strongly associated with *Ct* infection, present in 8.1% of cases compared to 2.4% of *Ct*-negative cases [9]. Nucleic Acid Amplification Tests (NAATs) or Polymerase Chain Reaction tests (PCRs) are reliable in the diagnosis of *Ct* and *Mg*, and in recent years, serological assays, among other methods, have been exploited to better understand the epidemiology of these infections [10].

Quantification of different *Ct* and *Mg* serum antibodies can provide an accurate means to evaluate past or ongoing infections [10–13]. Plasmid Gene Protein 3 (known as pgp3 or pGp3) is a highly immunogenic antigen during chlamydial infection [14–17], and notably, the antibodies produced against pGp3 exhibit a strong dependence on its protein conformation. In a UK-based study [13,18] that compared several assays in the same population, pGp3 serology was the most sensitive to detect a previously known *Ct* infection.

*M. genitalium* protein of adhesion (MgPa) is the major adhesin protein and the primary virulence factor of *Mg*, playing an essential role in mediating the attachment of the bacteria to host cells, thus facilitating their subsequent invasion [11,19,20]. Recombinant MgPa (rMgPa) is the C-terminal part of MgPa. Serological assays using MgPa and rMgPa antigens have shown promise in detecting *Mg* antibodies without cross-reactivity to *Mycoplasma pneumoniae* [11,21]. The interactions between *Mg* antigens and the host immune responses, however, remain largely unexplored.

The primary objective of this study was to cast further light on understanding the serological outcomes of *Ct* and *Mg* antibodies in young women and men and to exploit the possible co-factors role in *Ct* and *Mg* seropersistence.

## Material and methods

### Subjects

This study is based on the prospective Finnish HPV Family Study (FFHPV) cohort study jointly conducted at the Department of Obstetrics and Gynecology, Turku University Hospital (TUH), and the Institute of Dentistry, Faculty of Medicine, University of Turku, Finland [22]. The participants comprised 329 pregnant women and 135 fathers-to-be and their newborns who were enrolled (between 1998 and 2001) during the index pregnancy, at 36 weeks of pregnancy [22], and subsequently followed up for three years. The adult participants comprised spouses, however, the spouses of 194 women opted not to take part in the study, thus accounting for the difference in the number of male and female participants. All study participants were of Caucasian descent and shared the same ethnic background [23]. The Research Ethics Committee of Turku University Hospital has approved this study’s design (#3/1998 and 2/2006, with amendments 45/1801/2018).

The participants were requested to complete a structured questionnaire at baseline and repeated as shorter versions at 3-year and 6-year visits. It encompassed over 60 meticulously designed questions, including information about demographics, sexual behavior, smoking habits, history of STIs, and overall health. Responses about the childbirth experience were collected from the mothers at approximately two months post-partum. To minimize the potential bias, the spouses participating in this study were deliberately kept uninformed regarding the content of his/her spouse’s questionnaires [22].

### Serology

Blood samples were collected at the baseline and subsequently at 12-, 24- and 36-month follow-up visits from both spouses. The serological assays were performed in collaboration with the German Cancer Research Center (DKFZ), Heidelberg, Germany. For *Ct*, the serum IgG-antibodies to highly immunogenetic antigen pGp3 were assayed [14,15]. Serology to *Mg* was analyzed using the primary virulence factor of this bacterium, MgPa, in two protein fragments, MgPa N-Term, and rMgPa [19,20]. Studies about *Mg* serology are scanty, but antibodies targeting MgPa and rMgPa have been previously described [11,24]. The quantitative multiplex serology assay was used as previously described [24,25]. This method is based on glutathione S-transferase (GST) capture ELISA combined with fluorescent-bead technology. The median fluorescence intensity (MFI) of at least 100 beads per antigen was measured, and the cut-off values for *Ct* (pGp3) seropositivity was MFI > 500 as well as for *Mg* MFI > 1,000 for both MgPa N-Term and rMgPa. Assay development and validation are described in detail earlier [24].

### Statistical analysis

The flowchart of the study design is illustrated in Supplementary Figure 1. Overall, 280 women and 115 men in the *Ct* antibody analysis and 264 women and 113 men in the *Mg* antibody analysis were included in the final statistical analyses. They all had at least two visits during the follow-up. Subjects with only one blood sample or no visits were excluded (50 women and 20 men). Additionally, we excluded participants with fluctuating serological results, e.g., seropositive at baseline, negative at the next follow-up, and then again positive (1 woman and 4 men from the *Ct-* and 17 women and 6 men from the *Mg* analyses).

The final cohorts were divided into subgroups according to the seropersistence to *Ct* and/or *Mg.* The always negative subgroup consisted of participants whose antibody levels remained below the defined cut-off value at every follow-up visit. The seropersistent subgroup consisted of the participants, who remained constantly seropositive to the given antigen(s) during the entire follow-up. The seroconversion group included individuals whose antibody levels transitioned from seronegative to seropositive, with an additional criterion of at least a two-fold increase over their previous serum measurement, after which all subsequent antibody levels for these individuals remained consistently seropositive throughout the follow-up period. Respectively, the serological decay subgroup consisted of those participants whose antibody titers were falling at least 50% from the previous serum titer below the cut-off values and remained negative until the end of the follow-up. For *Mg*, both antigens (Mg-Pa and rMgPa) had to increase/decrease the above amount to be included in the final serology outcomes groups.

Frequency tables were analyzed using the χ^2^ test, employing the likelihood ratio or Fisher’s exact test as appropriate for categorical variables. For the examination of differences in means of continuous variables, nonparametric tests (Mann–Whitney U test or Kruskal-Wallis) test were used. Crude and adjusted odds ratios (ORs) and their 95% confidence intervals (95%CI) were calculated by using logistic regression. In the adjusted model, age and all baseline co-factors that were statistically significant in the crude analysis were simultaneously included for mutual adjustment. All statistical analyses were performed using STATA MP17.0 (Stata Corp., College Station, TX, USA). All tests were run two-sided, and, in all analyses, probability values (p-values) of <0.05 were considered statistically significant.

## Results

**Figure 1.**
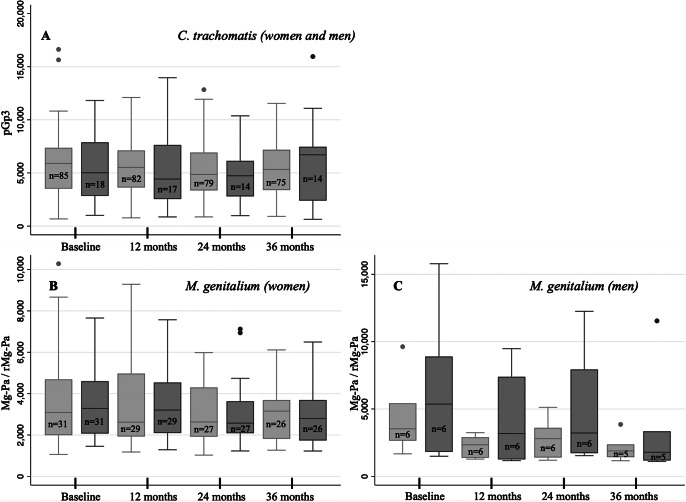
Mean IgG-antibody levels to *Chlamydia trachomatis* antigen pGp3 and *Mycoplasma genitalium* antigens MgPa and rMgPa among the seropersistent women and men followed for 3 years. (a) Mean antibody levels to *C. trachomatis* in the seropersistent subgroup stratified by follow-up visit. The light gray bars represent women, and the darker gray bars represent men; (b) Mean antibody levels to *M. genitalium* in women (lighter gray for MgPa and darker gray for rMgPa) and (c) Mean antibody levels to *M. genitalium* in men (lighter gray for MgPa and darker gray for rMgPa). n = stands for the number of seropositive individuals at each visit.

*Ct* seroprevalence at baseline was 32.4% among all women and 20.3% among all men. The corresponding results for *Mg* were 16.4% and 8.3%, respectively. The mean MFI antibody levels of pGp3 (*Ct*) and MgPa and rMgPa (*Mg)* among the seropersistent women and men stratified by their FU-visit are shown in Figure 1. The mean MFI levels of all three antibodies remained stable during the whole FU period of the seropersistent women. In men, however, variation of the levels of two IgG antibodies (MgPa and rMg) of *Mg* was seen, although the number of *Mg* seropositive men was much lower (only 5 to 6 men at each follow-up point).

The majority of both women and men remained seronegative to *Ct*, 65.7% and 81.7%, respectively. The same was true for *Mg*, to which 85.2% of women and 92.0% of men tested constantly seronegative. Persistent seropositivity to both *Ct* and *Mg* was more common in women (30.4% and 13.3%) than in men (17.4% and 5.3%), respectively. Seroconversion or serological decay were rare events. Additionally, there were only 20 women and 3 men who were persistently seropositive to *Ct* and *Mg* during the 3-year follow-up. Regarding the serological outcomes between couples, there were 72 couples in *Ct-* and 92 couples in *Mg* analyses with corresponding serological profiles, either seropositive or negative. These data are summarized in detail in Supplementary Table 1.

The demographic data stratified by the different serological outcomes of *Ct* and *Mg* among women are shown in Supplementary Table 2. The mean age of women varied between 23 to 27 years among the serological outcome groups. Women with persistent *Ct* antibodies were older than those always seronegative (26 years vs. 25 years, *p = 0.016*).

The potential co-factors associated with seropersistence for *Ct* and *Mg* in women during the follow-up were first evaluated with crude ORs (Supplementary Table 3), followed by Table 1, which has all significant baseline co-factors adjusted with each other. The increasing number of lifetime sexual partners was associated with persistent (pGp3) seropositivity to *Ct, as* the OR increased in parallel with the increasing number of lifetime sexual partners, up to crude OR 12.1 (95%CI 1.29–32.79) in those who reported more than 10 lifetime sex partners. This association stayed significant also with the adjusted model with the 6–10 and > 10 partners, with an OR of 5.5 and 5.6 (95%CI range of 1.39 to 22.29). Seropersistence to *Mg* was significantly associated only with more than 10 lifetime sex partners, crude OR 15.5 (95%CI 3.36–59.71). However, this did not stay significant after adjustment. Sexual debut at the age of 16 years or later was associated with lower odds for seropersistence to *Ct* and *Mg* separately or in combination, but after adjustment, none of these remained their significance. Women reporting more than 10 sexual partners before the age of 20 years had relations to becoming persistently seropositive to *Ct:* crude OR 5.2 (95%CI 1.82–14.72) or *Mg:* crude OR 14.6 (95%CI 3.57–59.71) or both: crude OR 27.7 (95%CI 4.18–182.92). Interestingly, after adjustment, only persistent *Mg* remained significant with an adjusted OR of 12.3 (95%CI 1.52–100.90).

**Table 1.** Age and all potential significant baseline co-factors^*^ adjusted with each other of persistent seropositivity versus seronegativity to *Chlamydia trachomatis* and/or *Mycoplasma genitalium* among *women* in the Finnish Family HPV study

	*C. trachomatis*	*M. genitalium*	Combination^†^
	Adjusted OR (95%Cl)		
**Questionnaire at baseline visit**			
Age	1.1 (0.96–1.19)	**1.2 (1.01–1.36)**	1.2 (0.94–1.45)
Smoking	0.8 (0.41–1.57)	1.6 (0.67–3.98)	1.2 (0.32–4.47)
Number of lifetime sex partners			
0–2	1.0	1.0	…
3–5	2.5 (0.88–7.37)	1.7 (0.31–9.41)	…
6–10	**5.5 (1.65–18.58)**	1.3 (0.21–8.75)	…
>10	**5.6 (1.39–22.29)**	2.8 (0.39–20.32)	…
Age at first sexual intercourse ≥16 years	0.6 (0.28–1.27)	0.8 (0.26–2.44)	0.5 (0.08–3.23)
Number of sex partners before the age of 20			
0–2	1.0	1.0	1.0
3–5	1.3 (0.53–2.30)	**5.0 (1.23–20.17)**	4.7 (0.42–43.03)
6–10	0.7 (0.21–2.34)	**6.2 (1.17–32.85)**	3.1 (0.25–39.08)
> 10	1.2 (0.25–6.04)	**12.3 (1.52–100.90)**	6.0 (0.25–147.95)
Practise of anal sex			
Never	1.0	1.0	1.00
Occasionally	**2.1 (1.00–4.58)**	1.9 (0.74–4.83)	3.0 (0.73–11.92)
Regularly	**15.3 (1.18–197.12)**	11.4 (0.80–162.57)	**168.8 (3.22–8,845.50)**
History of reported STIs			
None	1.0	1.0	1.00
Chlamydia	**18.0 (5.59–57.92)**	1.2 (0.37–4.12)	**16.1 (2.24–115.93)**
Genital herpes	1.9 (0.46–7.75)	1.2 (0.25–6.23)	4.0 (0.43–36.85)
Other/several STIs	**3.7 (1.15–11.90)**	1.2 (0.27–5.13)	1.4 (0.10–20.87)

* Crude ORs shown in Supplementary Table 3.

† Participants consistently testing negative/positive to both *C. trachomatis* and *M. genitalium*.

In women, occasional anal intercourse was connected to *Ct* seropositivity (crude OR 2.1, 95%CI 1.07–4.04) and with the *Mg* and *Ct* combination seropositivity (crude OR 3.4, 95%CI 1.14–10.12), while oral sex had no such association. After adjustment, the occasional and regular anal intercourse remained significant with *Ct* only, adjusted OR 2.1 (95%CI 1.00–4.58) and 15.3 (95%CI 1.18–197.12), respectively. As expected, reported STIs were associated with *Ct* seropersistence in both crude and adjusted models but did not influence *Mg* seropersistence. In our study, only 28.2% of the women and 45.0% of the men seropersistent to *Ct* reported a history of diagnosed infection.

Also, when evaluating factors at the end of the study follow-up, a higher number of deliveries seemed to be protective against seropersistence to *Ct.* There was nearly a 15-fold increased likelihood (95%CI 1.88–112.17) for seropersistence to *Ct* when the use of contraceptive pills was started at the age of 14 to 16 years. The association was also evident among women who had changed their partner since entering the cohort, with OR ranging from 2.7 (95%CI 1.24–6.05) to 4.9 (95%CI 1.44–16.52).

Table 2 and Supplementary Table 4 summarize the corresponding data of the men. For men, no separate adjusted OR table was done due to only one significant finding after adjustment, as further clarified under. The mean age varied between 27 and 36 years among the different serogroups of *Ct* and *Mg.* Men with 6–10 sex partners by the age of 20 years were more likely seropersistent to *Ct* (<5 as the reference group) crude OR of 3.4 (95%CI 1.05–11.14), but this did not stay significant after adjustment with overall sex partners. Men reporting >5 lifetime sexual partners were significantly associated with *Ct* seropersistence, which remained significant also after adjustment, adjusted OR 12.6 (95%CI 1.55–102.49). Importantly, men who reported having had several partners during the index pregnancy were more likely to have simultaneous seropositivity to both bacteria, crude OR 36.0 (95%CI 1.61–805.20). Interestingly, none of the co-factors included in our analysis showed any significant association with *Mg* seropersistence in men.

**Table 2.** Potential co-factors of persistent seropositivity versus seronegativity to *Chlamydia trachomatis* and/or *Mycoplasma genitalium* among men during the three-year follow-up. Significant crude associations highlighted in bold and those that remained significant after adjustment are underlined

	*C. trachomatis*	*M. genitalium*	Combination^a^
	Crude OR (95%Cl)		
**Questionnaire at baseline visit**			
Smoking	1.1 (0.40–3.27)	1.2 (0.19–7.63)	NC
Infertility	NC	NC	NC
> 5 sex partners overall	**13.7 (1.75–107.05)^b^**	NC	NC
≥ 5 sexual intercourses a month	0.9 (0.31–2.52)	1.0 (0.16–6.27)	0.6 (0.04–0.71)
Age at first sexual intercourse ≥16 years	0.4 (0.14–1.12)	0.6 (0.09–3.67)	NC
Number of sex partners before the age of 20			
0–5	1.0	1.0	1.0
6–10	**3.4 (1.05–11.14)**	1.6 (0.16–16.91)	…
> 10	1.8 (0.33–9.77)	3.1 (0.29–33.24)	…
Oral sex at least occasionally	0.6 (0.16–2.61)	…	…
Anal sex occasionally	1.5 (0.47–4.74)	6.2 (0.96–39.48)	4.4 (0.26–75.18)
History of reported STIs			
None	1.0	1.0	…
Chlamydia	**15.3 (2.46–95.19)**	1.7 (0.10–29.18)	…
Other	…	…	…
Allergies	1.8 (0.64–4.76)	2.2 (0.34–13.50)	1.7 (0.10–28.36)
Atopy	0.9 (0.10–8.08)	NC	NC
More than one sex partner during this pregnancy	2.5 (0.21–28.81)	11.6 (0.86–156.63)	**36.0 (1.61–805.20)**

a Participants consistently testing negative/positive to for both *C. trachomatis* and *M. genitalium*.

b Adjusted OR 12.6 (1.55–102.49).

As the female and male subjects of our study represent marital couples, we also made a pair-wise analysis of the serological outcomes among the seropersistent couples. However, only a limited number of couples demonstrated co-existent seropersistence for *Ct* (n = 10) or *Mg* (n = 4). As measured by the mean seropositivity among these couples, we could not disclose any significant correlations between the couples at any time point (using a scatterplot). As expected, the scatterplot yielded a limited number of measurements with notable dispersion. The same limitation hampered the analysis of the potential co-factors in these couples, including the meaningful calculation of odds ratios. Noteworthy, however, is the fact across all three categories (*Ct*, *Mg*, and antibody co-occurrence), none of the couples with seropersistence reported a history of five or fewer lifetime sexual partners (data not shown).

## Discussion

Our family study focused on the serological outcomes of *Ct* and *Mg* and also assessed the potential co-factors associated with their seropersistence, followed up for three years. The persistence of *Ct* antibodies was associated with a variety of sexual practices among women but less significantly among men. Among the women in our cohort, both adolescent and lifetime number of sexual partners were associated with both *Ct* and *Mg* seropersistence. Among the men in our study, no such associations were disclosed with *Mg* seropersistence. Until now, the majority of the risk factors for *Ct* and *Mg* have been focused on seropositivity, not seropersistence. However, it is important to understand i) whether the acquired IgG antibody levels remain stable, ii) which are the potential co-factors that predict the persistent seropositivity in a longitudinal setting, and iii) which are the possible consequences that might be attributed to seropersistence.

Previous studies have shown that both *Ct* and *Mg* are usually contracted during young adulthood. *Ct* usually peaks under the age of 25 in both genders [26] while *Mg* peaks in males at 25–34 years and in females at 16–19 years [4]. Microbe-specific serum IgG antibodies signify past or ongoing infection, though factors like antibody specificity, assay type, time since infection, and patient characteristics can affect IgG response and serological assay efficacy, particularly for prior *Ct* infections [18,27]. It is known that mucosal *Ct* infections typically elicit an observable rise in IgG responses when suitable serological assays are used. Thus, antibody levels can reflect both past or ongoing *Ct* infection [13,18,27,28], and in a clinical setting to confirm the infection, additional characterizations of the pathogen’s nucleic acids are needed.

In a Finnish study, *Ct* IgG antibodies to MOMP (chlamydial major outer membrane protein) were detected in 65.5% of women within three months of infection onset, with approximately one-third remaining seropositive for 3–10 years after the initial infection [29]. Another study of a female cohort using the same antigen as in the present study reported that pGp3 antibodies persisted for up to 12 years, and the antibody prevalence in the female cohort was higher than in their male counterparts [18]. Another study based on 9.695 biobank samples with a wider age distribution showed that pGp3 seroprevalence was 25.7% among women and 15.9% in men [30]. Our study confirms these observations on the stability of *Ct* antibodies and reports a similar prevalence of these antibodies (being higher in women), although the participants in our study were significantly younger.

As both *Ct* and *Mg* are STIs, we analyzed sexual practices, but also non-sexual co-factors as predictors of seropersistence. The present analysis revealed that an increasing number of lifetime sexual partners and early onset of sexual activities were significantly associated with seropersistence to both bacteria. Previous studies have confirmed the association between an increased number of sexual partners and the prevalence of *Ct* and *Mg.* In the study by Horner and colleagues [13], the OR increased in parallel with an increasing number of sexual partners, and in participants who reported having had 20 or more partners, OR for pGp3 seropositivity ranged from 49.1 to 58.7, depending on whether the participants had reported past *Ct* infection. Early sexual debut was also associated with seropositivity, consistent with our results. As for *Mg* infections [31], it was reported that their prevalence was more common in women reporting two or more sexual partners in the previous year (adjusted OR 2.2). In another survey, a strong association of *Mg* with sexual risk behaviors was found in both genders [32]. Noteworthy is the fact that these studies focused on NAAT detection, being different from our approach.

The prevalence of *Mg* infection closely parallels the prevalence of *Ct* among women with high-risk sexual behavior, such as multiple partners and inconsistent condom use [33]. Studies on simultaneous serology of *Ct* and *Mg* are scarce, and studies on seropersistence measured with different *Mg* antigens are nearly lacking. In our cohort, seropersistence to both pathogens was more common among women (11.1%) than men (3.5%). Persistence of *Mg* infection has been demonstrated before [8,31], although those studies used NAATs and had shorter follow-up periods compared to our serological approach. Additional adequately powered studies are needed to acquire a comprehensive view of *Mg* infections and their clinical implications. Given their shared STI pathogenesis, it is likely that the factors driving *Mg* transmission and serological persistence parallel those identified for *Ct.*

*Ct* infections are extensively studied due to their serious reproductive health consequences when untreated, including increased risks of adverse pregnancy and fertility outcomes [2,26]. Recently, it was shown that *Mg* seropositivity was also more common among women of infertile couples (5.4%) than fertile couples (1.6%) with an OR of 3.5 (95%CI 1.10–10.75) [12]. However, no such association was observed in male partners [12]. In our study, all women were pregnant at baseline, and only one fertility-associated co-factor could be identified; a higher parity was a protective factor against *Ct.* This likely reflects more stable relationships and reduced exposure to *Ct* due to fewer lifetime sexual partners and lower engagement in high-risk behaviors. Notably, among women with 10 or more lifetime sexual partners, having a parity of three or more was associated with a stronger protective effect, adjusted OR 0.3 (95%CI 0.09–0.89).

It can be speculated that protective factors against *Mg* seropersistence, including a lower number of sexual partners, could be similar to those of *Ct.* However, our study did not substantiate this hypothesis. A limitation of this study is the relatively small cohort size, which restricted our ability to explore the causes of seroconversion or decay in greater depth. Additionally, reliance on self-reported data for variables such as STI history, partner count, and sexual behaviors may have introduced potential non-differential misclassification, adding a degree of vulnerability to our findings. An important strength of our study is its longitudinal design, with repeated serum sampling throughout the follow-up period, allowing for a detailed analysis of changes over time.

Taken together, persistent *Ct* antibodies exhibit a strong association with well-defined high-risk sexual practices among women, less significantly in males. It can be concluded that appropriate preventive measures are of vital importance in combating STIs, irrespective of gender. Notably, seropositivity to *Mg* was predominantly correlated with the early sexual behavior of women but not those of men. Further studies of both genders are warranted to comprehensively assess the factors associated with *Mg* infection and the development of serological persistence.

## Supporting information

10.1017/S095026882500007X.sm001 Koskela et al. supplementary material Koskela et al. supplementary material

## Data Availability

The datasets used and/or analyzed during the current study are available from the corresponding author upon reasonable request.

## Supplementary material

The supplementary material for this article can be found at http://doi.org/10.1017/S095026882500007X.
