# Supplementary material for: Risk factors associated with IgG seropersistence to Chlamydia trachomatis and Mycoplasma genitalium
Source: Epidemiol Infect. 2025 Jan 24;153:e104. doi: 10.1017/S095026882500007X (PMC12455505; doi:10.1017/S095026882500007X)
Supplement: Koskela et al. supplementary material [file S095026882500007Xsup001.zip › S095026882500007Xsup002.docx]

**Supplementary Material**

| **Supplementary Table 1.** Serological outcomes of *C. trachomatis* and *M. genitalium* among the marital couples of the Finnish Family HPV study during the three-year follow-up ^a^. | | | | | | |
| --- | --- | --- | --- | --- | --- | --- |
|  |  | *Chlamydia trachomatis (pGp3)* | | *Mycoplasma genitalium (Mg/rMg)* | | *Combination ^b^* |
|  | | n (%) | MFI* mean (± SD) | n (%) | MFI** mean (± SD) | n (n%) |
| Women | | N=280 | | N=264 | | N=180 |
| Always negative ^c^ | | 184 (65.7) | 19.3 (3.3) | 225 (85.2) | 292.1 (28.5) / 474.7 (41.0) | 159 (88.3) |
| Seroconversion ^d^ | | 10 (3.6) | 2279.4 (586.4) | 4 (1.5) | 1658.3 (635.5) / 1968.4 (632.0) | 1 (0.6) |
| Always persistent ^e^ | | 85 (30.4) | 5474.9 (256.6) | 35 (13.3) | 3236.4 (268.3) / 3249.5 (245.9) | 20 (11.1) |
| Serological decay ^f^ | | 1 (0.4) | 438.0 (0.0) | 0 (0.0) | 0.0 (0.0) / 0.0 (0.0) | 0 (0.00) |
| Men |  | N=115 | | N=113 | | N=85 |
| Always negative ^c^ | | 94 (81.7) | 26.1 (5.1) | 104 (92.0) | 492.8 (84.5) / 454.1 (45.2) | 82 (96.5) |
| Seroconversion ^d^ | | 0 (0.00) | 0.0 (0.0) | 2 (1.8) | 777.7 (286.0) / 3695.5 (894.5) | 0 (0.0) |
| Always persistent ^e^ | | 20 (17.4) | 5475.6 (728.7) | 6 (5.3) | 2977.5 (560.5) / 5054.0 (1753.5) | 3 (3.5) |
| Serological decay ^f^ | | 1 (0.87) | 265.8 (0.0) | 1 (0.9) | 651.7 (0.0) / 1201.0 (0.0) | 0 (0.0) |
| Couples ^g^ | |  | N=72 | N=92 | | N=51 |
| Always negative ^c^ | | 62 (86.1) | 19.8 (3.2) | 88 (95.7) | 428.4 (52.2) / 461.9 (37.2) | 49 (96.1) |
| Seroconversion ^d^ | | 0 (0.0) | 0.0 (0.0) | 0 (0.0) | 0.0 (0.0) | 0 (0.0) |
| Always persistent ^e^ | | 10 (13.9) | 6286.2 (783.9) | 4 (4.3) | 3232.9 (873.5) / 5631.3 (921.8) | 2 (3.9) |
| Serological decay ^f^ | | 0 (0.0) | 0.0 (0.0) | 0 (0.0) | 0.0 (0.0) | 0 (0.0) |
| *^a^ Participants with at least two serum samples, fluctuating results excluded.*  *^b^ Participants consistently testing negative/positive for both CT and MG, and those that decayed or seroconverted to pathogens.*  *^c^ Participants whose antibody levels remained below the defined cut-off during the follow-up.*  *^d^ Participants whose antibody levels exhibited a two-fold increase surpassing the cut-off during the follow-up.*  *^e^ Participants whose antibody levels consistently surpassed the cut off value during the follow-up.*  *^f^ Participants whose antibody levels exhibited a 50% decline below the cut-off during the follow-up.*  ^g^ *Couples of the women and men form the FFHPV study that both had at least two serum samples during the follow-up and the same serological outcomes recorded.*  *Overall mean in 3 years, MFI cut-off >500  **Overall mean in 3 years, MFI cut-off>1000 | | | | | | |

| **Supplementary Table 2.** Demographic characteristics of the women from the Finnish Family HPV study ^a^ stratified by the serological outcome groups of *C. trachomatis* (n=280) and *M. genitalium* (n=264). | | | | | | | | | |
| --- | --- | --- | --- | --- | --- | --- | --- | --- | --- |
|  |  | *Chlamydia trachomatis* | | | | *Mycoplasma genitalium* | | | |
| Women |  | Always negative | Always persistent | Sero-conversion | Serological decay | Always negative | Always persistent | Sero-conversion | Serological decay |
| N (n%) |  | 184 (65.7) | 85 (30.3) | 10 (3.6) | 1 (0.4) | 225 (85.2) | 35 (13.3) | 4 (1.5) | – |
| Mean age (±SD) | | 25.05 (0.2) | 26.09 (0.4) | 25.90 (0.7) | 27.00 (0.0) | 25.27 (0.2) | 26.16 (0.6) | 23.25 (1.3) | – |
| Marital status n (n%) | |  |  |  |  |  |  |  |  |
|  | Single | 9 (5.2) | 9 (11.1) | 2 (20.0) | 0 (0.0) | 11 (5.2) | 6 (18.8) | 1 (25.0) | – |
|  | Cohabiting | 79 (45.9) | 33 (40.7) | 4 (40.0) | 0 (0.0) | 91 (42.9) | 12 (37.5) | 42 (50.0) | – |
|  | Married | 83 (48.3) | 38 (46.9) | 5 (40.0) | 1 (100.0) | 108 (50.9) | 14 (43.8) | 2 (25.0) | – |
|  | Divorced | 1 (0.6) | 1 (1.2) | 0 (0.0) | 0 (0.0) | 2 (0.9) | 0 (0.0) | 0 (0.0) | – |
| Education n (%) | |  |  |  |  |  |  |  |  |
|  | Compulsory school | 12 (7.0) | 9 (11.1) | 1 (10.0) | 0 (0.0) | 17 (8.0) | 4 (12.5) | 1 (25.0) | – |
|  | Vocational training | 48 (27.9) | 22 (27.2) | 1 (10.0) | 0 (0.0) | 55 (25.9) | 9 (28.1) | 1 (25.0) | – |
|  | Undergraduate | 33 (19.2) | 15 (18.5) | 2 (20.0) | 0 (0.0) | 40 (18.9) | 6 (18.8) | 0 (0.0) | – |
|  | College graduate | 51 (29.7) | 26 (32.1) | 4 (40.0) | 1 (100.0) | 64 (30.2) | 11(34.4) | 2 (50.0) | – |
|  | Academic degree | 28 (16.3) | 9 (11.1) | 2 (20.0) | 0 (0.0) | 36 (17.0) | 2 (6.23) | 0 (0.0) | – |
| Employment n (n%) | |  |  |  |  |  |  |  |  |
|  | Working | 99 (58.9) | 53 (67.1) | 3 (30.0) | 1 (100.0) | 129 (62.6) | 18 (56.3) | 1 (25.0) | – |
|  | Studying | 27 (16.1) | 12 (15.2) | 2 (20.0) | 0 (0.0) | 32 (15.5) | 4 (12.5) | 1 (25.0) | – |
|  | Unemployed | 42 (25.0) | 14 (17.7) | 5 (50.0) | 0 (0.0) | 45 (21.8) | 10 (31.3) | 2 (50.0) | – |
| Parity (n%) | |  |  |  |  |  |  |  |  |
|  | 0 | 1 (0.6) | 1 (1.2) | 0 (0.0) | 0 (0.0) | 2 (0.9) | 0 (0.0) | 0 (0.0) |  |
|  | 1 | 136 (79.1) | 51 (63.0) | 8 (80.0) | 0 (0.0) | 156 (73.6) | 23 (71.9) | 4 (100.0) | – |
|  | 2 | 31 (18.0) | 24 (29.6) | 2 (20.0) | 1 (100.0) | 49 (23.1) | 6 (18.8) | 0 (0.0) | – |
|  | 3 | 2 (1.2) | 4 (4.9) | 0 (0.0) | 0 (0.0) | 4 (1.9) | 1 (3.1) | 0 (0.0) | – |
|  | 4 | 2 (1.2) | 1 (1.2) | 0 (0.0) | 0 (0.0) | 1 (0.5) | 2 (6.3) | 0 (0.0) | – |
| Number of sex partners n (n%) | |  |  |  |  |  |  |  |  |
|  | 0–2 | 60 (35.1) | 6 (7.4) | 1 (10.0) | 0 (0.0) | 64 (30.3) | 2 (6.3) | 0 (0.0) | – |
|  | 3–5 | 58 (33.9) | 20 (24.7) | 5 (50.0) | 1 (100.0) | 69 (32.7) | 7 (21.9) | 3 (75.0) | – |
|  | 6–10 | 29 (17.0) | 26 (32.1) | 4 (40.0) | 0 (0.0) | 45 (21.3) | 7 (21.9) | 1 (25.0) | – |
|  | > 10 | 24 (14.0) | 29 (35.8) | 0 (0.0) | 0 (0.0) | 33 (15.6) | 16 (50.0) | 0 (0.0) | – |
| Number of sexual intercourses a month n (%) | | |  |  |  |  |  |  |  |
|  | 0–1 | 3 (1.7) | 2 (2.3) | 2 (20.0) | 0 (0.0) | 5 (2.4) | 1 (3.1) | 1 (25.0) | – |
|  | 2–4 | 53 (30.8) | 28 (34.6) | 4 (40.0) | 0 (0.0) | 70 (33.0) | 10 (31.3) | 0 (0.0) | – |
|  | 5–10 | 100 (58.1) | 38 (46.9) | 2 (20.0) | 1 (100.0) | 115 (54.2) | 13 (40.6) | 3 (75.0) |  |
|  | > 10 | 16 (9.30) | 13 (16.05) | 2 (20.00) | 0 (0.00) | 22 (10.38) | 8 (25.0) | 0 (0.0) | – |
| Age (in years) at first sexual intercourse n (%) | |  |  |  |  |  |  |  |  |
|  | ≤ 13 | 3 (1.7) | 4 (4.9) | 0 (0.0) | 0 (0.0) | 6 (2.8) | 0 (0.0) | 0 (0.0) | – |
|  | 14–16 | 87 (50.6) | 56 (69.1) | 5 (50.0) | 1 (100.0) | 111 (52.4) | 25 (78.1) | 4 (100.0) | – |
|  | 17–19 | 71 (41.3) | 20 (24.7) | 5 (50.0) | 0 (0.0) | 86 (40.6) | 7 (21.9) | 0 (0.0) | – |
|  | ≥ 20 | 11 (6.4) | 1 (1.2) | 0 (0.0) | 0 (0.0) | 9 (4.3) | 0 (0.0) | 0 (0.0) |  |
| Number of sex partners before the age of 20 n (%) | | |  |  |  |  |  |  |  |
|  | 0–2 | 87 (50.6) | 21 (25.9) | 6 (60.0) | 0 (0.00) | 107 (50.5) | 4 (12.5) | 0 (0.0) | – |
|  | 3–5 | 54 (31.4) | 32 (39.5) | 4 (40.0) | 1 (100.0) | 66 (31.1) | 13 (40.6) | 3 (75.0) | – |
|  | 6–10 | 23 (13.4) | 18 (22.2) | 0 (0.0) | 0 (0.0) | 28 (13.2) | 9 (28.1) | 1 (25.0) | – |
|  | > 10 | 8 (4.7) | 10 (12.4) | 0 (0.0) | 0 (000) | 11 (5.2) | 6 (18.8) | 0 (0.0) | – |
| Practise of oral sex n (%) | |  |  |  |  |  |  |  |  |
|  | Regularly | 18 (10.5) | 11 (13.6) | 3 (30.0) | 0 (0.0) | 24 (11.3) | 5 (15.6) | 1 (25.0) |  |
|  | Occasionally | 115 (66.7) | 58 (71.6) | 3 (30.0) | 1 (100.0) | 140 (66.0) | 22 (68.8) | 3 (75.0) | – |
|  | Never | 39 (22.7) | 12 (14.8) | 4 (40.0) | 0 (0.0) | 48 (22.6) | 5 (15.6) | 0 (0.0) | – |
| Practise of anal sex n (%) | |  |  |  |  |  |  |  |  |
|  | Regularly | 1 (0.6) | 2 (2.5) | 0 (0.0) | 0 (0.0) | 2 (0.9) | 1 (3.1) | 0 (0.0) | – |
|  | Occasionally | 24 (14.0) | 20 (24.7) | 4 (40.0) | 0 (0.0) | 35 (16.5) | 9 (28.1) | 1 (25.0) | – |
|  | Never | 147 (85.5) | 59 (72.8) | 6 (60.0) | 1 (100.0) | 175 (82.6) | 22 (68.8) | 3 (75.0) | – |
| History of reported STIs n (%) | |  |  |  |  |  |  |  |  |
|  | Chlamydia | 4 (2.2) | 24 (28.2) | 1 (10.0) | 0 (0.0) | 20 (8.9) | 6 (17.1) | 0 (0.00) | – |
|  | Genital herpes | 5 (2.7) | 5 (5.9) | 0 (0.0) | 0 (0.0) | 7 (3.1) | 3 (8.6) | 0 (0.00) | – |
|  | Other / several STIs | 6 (3.3) | 10 (11.8) | 1 (10.0) | 0 (0.0) | 10 (4.4) | 3 (8.6) | 1 (25.0) | – |
|  | None | 169 (91.9) | 46 (54.1) | 8 (80.0) | 1 (100.0) | 188 (83.6) | 23 (65.7) | 3 (75.0) | – |
| Starting age of contraceptive pills n (%) | |  |  |  |  |  |  |  |  |
|  | ≤ 13 years | 2 (1.2) | 1 (1.2) | 0 (0.0) | 0 (0.0) | 3 (1.4) | 0 (0.0) | 0 (0.0) | – |
|  | 14–16 years | 62 (36.1) | 45 (55.6) | 3 (30.0) | 0 (0.0) | 76 (35.9) | 20 (62.5) | 2 (50.0) | – |
|  | 17–19 years | 71 (41.3) | 27 (33.3) | 5 (50.0) | 1 (100.0) | 91 (42.9) | 9 (28.1) | 1 (25.0) | – |
|  | ≥ 20 years | 17 (9.9) | 7 (8.6) | 1 (10.0) | 0 (0.0) | 23 (10.9) | 1 (3.1) | 0 (0.0) | – |
|  | Never | 20 (11.6) | 1 (1.2) | 1 (10.0) | 0 (0.0) | 19 (9.0) | 2 (6.3) | 1 (25.0) | – |
| Smoking n (%) | |  |  |  |  |  |  |  |  |
|  | Yes | 80 (46.5) | 47 (58.0) | 5 (50.0) | 1 (100.0) | 98 (46.2) | 22 (68.8) | 2 (50.0) | – |
|  | No | 92 (53.5) | 34 (42.0) | 5 (50.0) | 0 (0.0) | 114 (53.8) | 10 (31.3) | 2 (50.0) | – |
| Alcohol comsumption n (%) | |  |  |  |  |  |  |  |  |
|  | Never | 22 (12.8) | 5 (6.3) | 1 (10.0) | 0 (0.0) | 24 (11.4) | 3 (9.4) | 0 (0.0) | – |
|  | One dose a month | 79 (45.9) | 42 (52.5) | 5 (50.0) | 0 (0.0) | 106 (50.2) | 14 (43.8) | 2 (50.0) | – |
|  | One dose a week | 50 (29.1) | 27 (33.8) | 3 (30.0) | 1 (100.0) | 59 (28.0) | 13 (40.6) | 2 (50.0) | – |
|  | One dose 2–3 times a week | 20 (11.6) | 6 (7.5) | 1 (10.0) | 0 (0.0) | 21 (10.0) | 2 (6.3) | 0 (0.0) | – |
|  | One dose a day | 1 (0.6) | 0 (0.0) | 0 (0.0) | 0 (0.0) | 1 (0.5) | 0 (0.00) | 0 (0.00) | – |
| Allergies n (n%) | |  |  |  |  |  |  |  |  |
|  | Yes | 76 (44.4) | 36 (45.0) | 3 (30.0) | 1 (100.0) | 93 (44.3) | 14 (43.8) | 3 (75.0) | – |
|  | No | 95 (55.6) | 44 (55.0) | 7 (70.0) | 0 (0.0) | 117 (55.7) | 18 (56.3) | 1 (25.0) | – |
| Atopy n (%) |  |  |  |  |  |  |  |  | – |
|  | Yes | 28 (16.7) | 12 (15.6) | 2 (20.0) | 0 (0.0) | 33 (16.0) | 8 (25.8) | 0 (0.0) | – |
|  | No | 140 (83.3) | 65 (84.4) | 8 (80.0) | 1 (100.0) | 173 (84.0) | 23 (74.2) | 4 (100.0) | – |
| Vaginitis during pregnancy n (%) | | |  |  |  |  |  |  |  |
|  | Yes, once | 21 (12.6) | 13 (16.2) | 3 (30.0) | 0 (0.0) | 27 (13.0) | 6 (19.6) | 2 (50.0) | – |
|  | Yes, twice | 2 (1.2) | 4 (5.0) | 0 (0.0) | 0 (0.0) | 4 (1.9) | 2 (6.6) | 0 (0.0) | – |
|  | Yes, more than two times | 2 (1.2) | 0 (0.0) | 0 (0.0) | 0 (0.0) | 2 (1.0) | 0 (0.0) | 0 (0.0) | – |
|  | None / not treated | 142 (85.0) | 63 (78.8) | 7 (70.0) | 1 (100.0) | 174 (84.0) | 23 (74.2) | 2 (50.0) | – |
| Number of sex partners during baseline pregnancy n (%) | | | |  |  |  |  |  |  |
|  | 1 partner | 169 (98.8) | 80 (100.0) | 10 (100.0) | 1 (100.0) | 209 (99.0) | 31 (100.0) | 4 (100.0) | – |
|  | > 1 partner | 2 (1.2) | 0 (0.0) | 0 (0.0) | 0 (0.0) | 2 (1.0) | 0 (0.0) | 0 (0.0) | – |
| Beginning of labor n (n%) | |  |  |  |  |  |  |  |  |
|  | Own contractions | 79 (47.6) | 39 (55.7) | 5 (50.0) | 0 (0.0) | 102 (51.0) | 17 (60.7) | 0 (0.0) | – |
|  | Breaking of the amniotic fluid | 33 (19.9) | 10 (14.3) | 0 (0.0) | 0 (0.0) | 37 (18.3) | 1 (3.6) | 0 (0.0) | – |
|  | Prostaglandins | 12 (7.2) | 8 (11.4) | 1 (10.0) | 0 (0.0) | 14 (6.9) | 4 (14.3) | 1 (33.3) | – |
|  | Oxytocin | 29 (17.5) | 7 (10.0) | 3 (30.0) | 0 (0.0) | 33 (16.3) | 4 (14.3) | 2 (66.7) | – |
|  | Rupturing of membranes | 4 (2.4) | 3 (4.3) | 0 (0.0) | 0 (0.0) | 7 (3.5) | 0 (0.0) | 0 (0.0) | – |
|  | Several manners | 9 (5.4) | 3 (4.3) | 1 (10.0) | 0 (0.0) | 9 (4.5) | 2 (7.1) | 0 (0.0) | – |
| Childbirth weeks of pregnancy n (%) | | | |  |  |  |  |  |  |
|  | < 40 weeks | 71 (38.6) | 32 (37.7) | 3 (30.0) | 1 (100.0) | 82 (36.4) | 17 (48.6) | 1 (25.0) | – |
|  | ≥ 40 weeks | 113 (61.4) | 53 (62.4) | 7 (70.0) | 0 (0.0) | 143 (63.6) | 18 (51.4) | 3 (75.0) | – |
| Birth weight of the newborn n (n%) | | |  |  |  |  |  |  |  |
|  | < 3000 g | 17 (9.2) | 6 (7.1) | 1 (10.0) | 0 (0.0) | 18 (8.0) | 3 (8.6) | 0 (0.0) | – |
|  | ≥ 3000 g | 167 (90.8) | 79 (92.9) | 9 (90.0) | 1 (100.0) | 207 (92.0) | 32 (91.4) | 4 (100.0) | – |
| *^a^ Participants completed the questionnaire at the first appointment. Information about delivery was collected at 2 months post-partum.* | | | | | | | | | |

| **Supplementary Table 3.** Potential co-factors of persistent seropositivity versus seronegativity to *C. trachomatis* and/or *M. genitalium* among *women* in the Finnish Family HPV study. | | | | | | | |
| --- | --- | --- | --- | --- | --- | --- | --- |
|  | | | | | *Chlamydia trachomatis* | *Mycoplasma genitalium* | *Combination ^a^* |
|  | | | | | Crude OR (95%Cl) | | |
| **Questionnaire at baseline visit**  Smoking | | | | | 1.6 (0.93–2.71) | **2.6 (1.16–5.67)** | 2.4 (0.90–6.46) |
| Infertility | | | | | 2.3 (0.87–6.03) | 0.8 (0.18–3.71) | 2.0 (0.12–8.29) |
| Number of miscarriages | | | | |  |  |  |
|  | | 0 | | | 1.00 | 1.0 | 1.0 |
|  | | 1 | | | 2.0 (0.89–4.32) | 0.6 (0.12–2.46) | 1.3 (0.27–6.44) |
|  | | ≥2 | | | 1.8 (0.39–8.32) | … | … |
| Number of lifetime sex partners | | | | | |  |  |
|  | | 0–2 | | | 1.0 | 1.0 | … |
|  | | 3–5 | | | **3.4 (1.29–9.20)** | 3.2 (0.65–16.21) | … |
|  | | 6–10 | | | **9.0 (3.32–24.18)** | 5.0 (0.99–25.08) | … |
|  | | >10 | | | **12.1 (4.45–32.79)** | **15.5 (3.36–71.57)** | … |
| ≥ 5 sexual intercourses a month | | | | | 0.8 (0.47–1.43) | 1.0 (0.48–2.28) | 1.1 (0.39–0.05) |
| Age at first sexual intercourse ≥ 16 years | | | | | **0.4 (0.22–0.69)** | **0.3 (0.14–0.83)** | **0.2 (0.05–0.66)** |
| Number of sex partners before the age of 20 | | | | |  |  |  |
|  | | 0–2 | | | 1.0 | 1.0 | 1.0 |
|  | | 3–5 | | | **2.5 (1.29–4.69)** | **5.3 (1.65–16.84)** | **6.8 (1.34–33.93)** |
|  | | 6–10 | | | **3.2 (1.49–7.07)** | **8.6 (2.47–29.98)** | **14.6 (2.72–78.84)** |
|  | | > 10 | | | **5.2 (1.82–14.72)** | **14.6 (3.57–59.71)** | **27.7 (4.18–182.92)** |
| Practise of oral sex | | | | |  |  |  |
|  | | Never | | | 1.0 | 1.0 | 1.0 |
|  | | Occasionally | | | 1.64 (0.80–3.37) | 1.51 (0.54–4.20) | 2.64 (0.57–12.19) |
|  | | Regularly | | | 1.99 (0.74–5.35) | 2.00 (0.53–7.58) | 3.96 (0.60–26.29) |
| Practise of anal sex | | | | |  |  |  |
|  | | Never | | | 1.0 | 1.0 | 1.0 |
|  | | Occasionally | | | **2.1 (1.07–4.04)** | 2.0 (0.87–4.82) | **3.4 (1.14–10.12)** |
|  | | Regularly | | | 5.0 (0.44–56.00) | 4.0 (0.35–45.68) | 10.8 (0.63–182.93) |
| History of reported STIs | | | | |  |  |  |
|  | | None | | | 1.0 | 1.0 | 1.0 |
|  | | Chlamydia | | | **22.0 (7.28–66.72)** | 2.5 (0.89–6.73) | **29.6 (6.43–136.25)** |
|  | | Genital herpes | | | **3.7 (1.02–13.24)** | 3.5 (0.85–14.49) | **8.9 (1.85–42.60)** |
|  | | Other / several STIs | | | **6.1 (2.11–17.73)** | 2.5 (0.63–9.56) | 4.9 (0.47–51.83) |
| Starting age (in years) of contraceptive pills | | | | |  |  |  |
|  | | Never used | | | 1.0 | 1.0 | … |
|  | | ≤ 13 | | | 10.0 (0.44–228.70) | … | … |
|  | | 14–16 | | | **14.5 (1.88–112.17)** | 2.5 (0.54–11.64) | … |
|  | | 17–19 | | | 7.6 (0.97–59.48) | 0.9 (0.19–4.70) | … |
|  | | ≥ 20 | | | 8.2 (0.92–73.79) | 0.4 (0.03–4.91) | … |
| Allergies | | | | | 1.0 (0.60–1.74) | 1.0 (0.46–2.07) | 0.6 (0.22–1.73) |
| Atopy | |  | | | 0.9 (0.44–1.93) | 1.8 (0.75–4.42) | 1.1 (0.30–4.22) |
| Labour start with water breaking | | | | | 0.7 (0.31–1.45) | 0.2 (0.02–1.25) | NC |
| Labour ≥40 weeks of pregnancy | | | | | 1.0 (0.61–1.77) | 0.6 (0.30–1.24) | 0.6 (0.24–1.58) |
| Birth weight of the newborn <3000g | | | | | 1.3 (0.51–3.53) | 0.9 (0.26–3.33) | 0.8 (0.17–3.84) |
| ≥1 vaginitis during this pregnancy | | | | | 1.5 (0.77–3.04) | 1.8 (0.76–4.45) | 2.9 (0.98–8.46) |
|  | | | | |  |  |  |
| **Questionnaire at final visit** | | | | |  |  |  |
|  | | | | |  |  |  |
| Different partner than at the baseline | | | | | **2.7 (1.24–6.05)** | **2.7 (1.07–6.98)** | **4.9 (1.44–16.52)** |
| Number of sexual intercourses a month | | | | |  |  |  |
|  | | | 0–1 | | 1.0 | 1.0 | 1.0 |
|  |  | | 2–4 |  | 0.8 (0.27–2.59) | 1.2 (0.25–6.20) | 1.5 (0.16–13.75) |
|  |  | | 5–10 |  | 0.6 (0.17–1.93) | 1.2 (0.21–6.34) | 1.2 (0.12–12.14) |
|  |  | | > 10 |  | 1.5 (0.34–6.53) | 1.8 (0.25–12.60) | 3.0 (0.22–40.93) |
| Parity | | | | |  |  |  |
|  | | | 1 | | 1.0 | 1.0 | 1.0 |
|  | | | 2 | | 0.5 (0.19–1.07) | 0.5 (0.17–1.51) | 0.3 (0.07–1.14) |
|  | | | ≥3 | | **0.3 (0.11–0.88)** | 0.6 (0.17–2.18) | 0.3 (0.05–1.45) |
| Current contraception | | |  | |  |  |  |
|  | | | No contraception | | 1.0 | 1.0 | 1.0 |
|  | | | Condom | | 1.4 (0.47–3.92) | 1.1 (0.27–4.22) | 0.6 (0.08–3.80) |
|  | | | Other | | 1.4 (0.55–3.42) | 1.1 (0 .06–0.53) | 1.0 (0.23–3.97) |
| *^a^ Participants consistently testing negative/positive to both Chlamydia trachomatis and Mycoplasma genitalium.* | | | | | | | |

| **Supplementary Table 4.** Background characteristics of men from the Finnish Family HPV study ^a^ stratified by the serological outcome groups of *C. trachomatis* (n=115) and *M. genitalium* (n=113). | | | | | | | | | |
| --- | --- | --- | --- | --- | --- | --- | --- | --- | --- |
|  |  | *Chlamydia trachomatis* | | | | *Mycoplasma genitalium* | | | |
| Men |  | Always negative | Always persistent | Sero-conversion | Serological decay | Always negative | Always persistent | Sero-conversion | Serological decay |
| N (n%) |  | 94 (81.7) | 20 (17.4) | – | 1 (0.9) | 104 (92.0) | 6 (5.3) | 2 (1.8) | 1 (1.0) |
| Mean age (±SD) | | 28.18 (0.5) | 30.58 (1.3) | – | 36.00 (0.0) | 28.72 (0.5) | 27.00 (1.9) | 26.50 (1.5) | 36.00 (0.0) |
| Marital status n (n%) | |  |  |  |  |  |  |  |  |
|  | Single | 1 (1.1) | 0 (0.0) | – | 0 (0.0) | 0 (0.0) | 1 (20.0) | 0 (0.0) | 0 (0.0) |
|  | Cohabiting | 35 (39.3) | 9 (47.34) | – | 1 (100.0) | 42 (42.4) | 1 (20.0) | 1 (50.0) | 1 (100.0) |
|  | Married | 52 (58.4) | 10 (52.6) | – | 0 (0.0) | 56 (56.6) | 3 (60.0) | 1 (50.0) | 0 (0.0) |
|  | Divorced | 1 (1.1) | 0 (0.0) | – | 0 (0.0) | 1 (1.0) | 0 (0.0) | 0 (0.0) | 0 (0.0) |
| Education n (%) | |  |  |  |  |  |  |  |  |
|  | Compulsory school | 7 (8.0) | 1 (5.3) | – | 0 (0.0) | 6 (6.1) | 1 (20.0) | 0 (0.0) | 0 (0.0) |
|  | Vocational training | 35 (39.8) | 10 (52.6) | – | 1 (100.0) | 42 (42.9) | 2 (40.0) | 2 (100.0) | 1 (100.0) |
|  | Undergraduate | 9 (10.2) | 1 (5.3) | – | 0 (0.0) | 8 (8.2) | 1 (20.0) | 0 (0.0) | 0 (0.0) |
|  | College graduate | 23 (26.1) | 6 (31.6) | – | 0 (0.0) | 28 (28.6) | 1 (20.0) | 0 (0.0) | 0 (0.0) |
|  | Academic degree | 14 (15.9) | 1 (5.3) | – | 0 (0.0) | 14 (14.3) | 0 (0.0) | 0 (0.0) | 0 (0.0) |
| Employment n (n%) | |  |  |  |  |  |  |  |  |
|  | Working | 76 (86.4) | 16 (89.0) | – | 1 (100.0) | 83 (85.6) | 5 (100.0) | 2 (100.0) | 1 (100.0) |
|  | Studying | 7 (8.0) | 1 (5.6) | – | 0 (0.0) | 8 (8.2) | 0 (0.0) | 0 (0.0) | 0 (0.0) |
|  | Unemployed | 5 (5.7) | 1 (5.6) | – | 0 (0.0) | 6 (6.2) | 0 (0.0) | 0 (0.0) | 0 (0.0) |
| Children from all relationships n (%) | | |  |  |  |  |  |  |  |
|  | 1 | 48 (57.8) | 9 (50.0) | – | 1 (100.0) | 53 (57.6) | 3 (60.0) | 0 (0.0) | 0 (0.0) |
|  | 2 | 23 (27.7) | 4 (22.2) | – | 0 (0.0) | 23 (25.0) | 2 (40.0) | 1 (50.0) | 1 (100.0) |
|  | 3 | 7 (8.4) | 3 (16.7) | – | 0 (0.0) | 10 (10.9) | 0 (0.0) | 1 (50.0) | 0 (0.0) |
|  | 4 | 3 (3.6) | 1 (5.6) | – | 0 (0.0) | 3 (3.3) | 0 (0.0) | 0 (0.0) | 0 (0.0) |
|  | 5 | 1 (1.2) | 1 (5.6) | – | 0 (0.0) | 2 (2.2) | 0 (0.0) | 0 (0.0) | 0 (0.0) |
|  | 6 | 1 (1.2) | 0 (0.0) | – | 0 (0.0) | 1 (1.1) | 0 (0.0) | 0 (0.0) | 0 (0.0) |
| History of infertility n (n%) | |  |  |  |  |  |  |  |  |
|  | No | 78 (89.7) | 19 (100.0) | – | 1 (100.0) | 91 (93.8) | 5 (100.0) | 1 (50.0) | 1 (100.0) |
|  | Yes | 9 (10.3) | 0 (0.0) | – | 0 (0.0) | 6 (6.2) | 0 (0.0) | 1 (50.0) | 0 (0.0) |
| Number of sex partners n (n%) | |  |  |  |  |  |  |  |  |
|  | 0–2 | 16 (18.2) | 0 (0.0) | – | 0 (0.0) | 12 (15.3) | 0 (0.0) | 0 (0.0) | 0 (0.0) |
|  | 3–5 | 22 (25.0) | 1 (5.3) | – | 1 (100.0) | 23 (23.5) | 0 (0.0) | 0 (0.0) | 0 (0.0) |
|  | 6–10 | 20 (22.7) | 6 (31.6) |  | 0 (0.0) | 21 (21.4) | 2 (40.0) | 0 (0.0) | 1 (100.0) |
|  | > 10 | 30 (34.1) | 12 (63.2) | – | 0 (0.0) | 39 (39.8) | 3 (60.0) | 2 (10.0) | 0 (0.0) |
| Number of sexual intercourses a month n (%) | | |  |  |  |  |  |  |  |
|  | 0–1 | 1 (1.2) | 0 (0.0) | – | 0 (0.0) | 1 (1.1) | 0 (0.0) | 0 (0.0) | 0 (0.0) |
|  | 2–4 | 30 (34.9) | 7 (39.0) | – | 1 (100.0) | 37 (39.0) | 2 (40.0) | 0 (0.0) | 0 (0.0) |
|  | 5–10 | 42 (48.8) | 9 (50.0) | – | 0 (0.0) | 44 (46.3) | 1 (20.0) | 2 (100.0) | 1 (100.0) |
|  | > 10 | 13 (15.1) | 2 (11.1) | – | 0 (0.0) | 13 (13.7) | 2 (40.0) | 0 (0.0) | 0 (0.0) |
| Age (in years) at first sexual intercourse n (%) | | |  |  |  |  |  |  |  |
|  | ≤ 13 | 1 (1.1) | 2 (10.5) | – | 0 (0.0) | 3 (3.1) | 0 (0.0) | 0 (0.0) | 0 (0.0) |
|  | 14–16 | 35 (39.8) | 10 (52.6) | – | 0 (0.0) | 43 (43.9) | 3 (60.0) | 0 (0.0) | 0 (0.0) |
|  | 17–19 | 41 (46.6) | 6 (31.6) | – | 1 (100.0) | 42 (42.9) | 2 (40.0) | 2 (100.0) | 1 (100.0) |
|  | ≥ 20 | 11 (12.5) | 1 (5.3) | – | 0 (0.0) | 10 (10.2) | 0 (0.0) | 0 (0.0) | 0 (0.0) |
| Number of sex partners before the age of 20  n (%) | | |  |  |  |  |  |  |  |
|  | 0–2 | 42 (48.3) | 4 (21.1) | – | 1 (100.0) | 43 (44.3) | 0 (0.0) | 0 (0.0) | 0 (0.0) |
|  | 3–5 | 27 (31.0) | 7 (36.8) | – | 0 (0.0) | 31 (32.0) | 3 (60.0) | 1 (50.0) | 1 (100.0) |
|  | 6–10 | 11 (12.6) | 6 (31.6) | – | 0 (0.0) | 15 (15.5) | 1 (20.0) | 1 (50.0) | 0 (0.0) |
|  | > 10 | 7 (8.1) | 2 (10.5) | – | 0 (0.0) | 8 (8.3) | 1 (20.0) | 0 (0.0) | 0 (0.0) |
| Practise of oral sex n (%) | |  |  |  |  |  |  |  |  |
|  | Regularly | 25 (28.4) | 3 (16.7) | – | 0 (0.0) | 24 (24.7) | 1 (20.0) | 2 (100.0) | 1 (100.0) |
|  | Occasionally | 53 (60.2) | 12 (66.7) | – | 0 (0.0) | 59 (60.8) | 4 (80.0) | 0 (0.0) | 0 (0.0) |
|  | Never | 10 (11.4) | 3 (16.7) | – | 1 (100.0) | 14 (14.4) | 0 (0.0) | 0 (0.0) | 0 (0.0) |
| Practise of anal sex n (%) | |  |  |  |  |  |  |  |  |
|  | Regularly | 0 (0.0) | 0 (0.0) | – | 0 (0.0) | 0 (0.0) | 0 (0.0) | 0 (0.0) | 0 (0.0) |
|  | Occasionally | 18 (20.5) | 5 (27.8) | – | 0 (0.0) | 19 (19.6) | 3 (60.0) | 0 (0.0) | 0 (0.0) |
|  | Never | 70 (79.5) | 13 (72.2) | – | 1 (100.0) | 78 (80.4) | 2 (40.0) | 2 (100.0) | 1 (100.0) |
| History of reported STIs n (%) | |  |  |  |  |  |  |  |  |
|  | Chlamydia | 5 (16.7) | 9 (81.8) | – | 0 (0.0) | 12 (30.0) | 1 (50.0) | 0 (0.0) | 0 (0.0) |
|  | Gonorrhea | 1 (3.3) | 0 (0.0) |  | 0 (0.0) | 1 (2.5) | 0 (0.0) | 0 (0.0) | 0 (0.0) |
|  | Genital herpes | 3 (10.0) | 0 (0.0) | – | 0 (0.0) | 3 (7.5) | 0 (0.0) | 0 (0.0) | 0 (0.0) |
|  | Other / several STIs | 4 (13.3) | 0 (0.0) | – | 0 (0.0) | 4 (10.0) | 0 (0.0) | 1 (100.0) | 0 (0.0) |
|  | None | 17 (56.7) | 2 (18.2) | – | 0 (0.0) | 20 (50.0) | 1 (50.0) | 0 (0.0) | 3 (60.0) |
| Smoking n (%) | |  |  |  |  |  |  |  |  |
|  | Yes | 31 (35.6) | 7 (38.9) | – | 0 (0.0) | 34 (35.4) | 2 (40.0) | 1 (50.0) | 1 (100.0) |
|  | No | 56 (64.4) | 11 (61.1) | – | 1 (100.0) | 62 (64.6) | 3 (60.0) | 1 (50.0) | 0 (0.0) |
| Alcohol comsumption n (%) | |  |  |  |  |  |  |  |  |
|  | Never | 2 (2.3) | 0 (0.0) | – | 0 (0.0) | 2 (2.1) | 0 (0.0) | 0 (0.0) | 0 (0.0) |
|  | One dose a month | 16 (18.4) | 7 (36.8) | – | 0 (0.0) | 23 (23.7) | 0 (0.0) | 0 (0.0) | 0 (0.0) |
|  | One dose a week | 33 (37.9) | 7 (36.8) | – | 1 (100.0) | 36 (37.1) | 2 (40.0) | 0 (0.0) | 1 (100.0) |
|  | One dose 2–3 times a week | 33 (37.9) | 5 (26.3) | – | 0 (0.0) | 33 (34.0) | 3 (60.0) | 2 (100.0) | 0 (0.0) |
|  | One dose a day | 3 (3.5) | 0 (0.0) | – | 0 (0.0) | 3 (3.1) | 0 (0.0) | 0 (0.0) | 0 (0.0) |
| Allergies n (n%) | |  |  |  |  |  |  |  |  |
|  | Yes | 33 (38.8) | 10 (52.6) | – | 1 (100.0) | 39 (41.1) | 3 (60.0) | 1 (50.0) | 1 (100.0) |
|  | No | 52 (61.2) | 9 (47.4) | – | 0 (0.0) | 56 (59.0) | 2 (40.0) | 1 (50.0) | 0 (0.0) |
| Atopy n (%) |  |  |  |  |  |  |  |  |  |
|  | Yes | 5 (5.9) | 1 (5.3) | – | 0 (0.0) | 8 (8.4) | 0 (0.0) | 0 (0.0) | 0 (0.0) |
|  | No | 80 (94.1) | 18 (94.7) | – | 1 (100.0) | 87 (91.6) | 5 (100.0) | 2 (100.0) | 1 (100.0) |
| Number of sex partners during this pregnancy n (%) | | | |  |  |  |  |  |  |
|  | 1 partner | 84 (97.7) | 17 (94.4) | – | 1 (100.0) | 93 (97.9) | 4 (80.0) | 2 (100.0) | 1 (100.0) |
|  | > 1 partner | 2 (2.3) | 1 (5.6) | – | 0 (0.0) | 2 (2.1) | 1 (20.0) | 0 (0.0) | 0 (0.0) |
| *^a^ Participants completed the questionnaire at the first appointment.* | | | | | | | | | |

**Supplementary Figure Legends**

**Supplementary Figure 1.** Flowchart of the couples in the Finnish Family HPV study who were eligible for the serological assays of *Chlamydia trachomatis* (*Ct*) and *Mycoplasma genitalium* (*Mg*) IgG-antibodies during the three-year follow-up.
