# Supplementary figures and images for: Risk factors associated with IgG seropersistence to Chlamydia trachomatis and Mycoplasma genitalium
Source: Epidemiol Infect. 2025 Jan 24;153:e104. doi: 10.1017/S095026882500007X (PMC12455505; doi:10.1017/S095026882500007X)

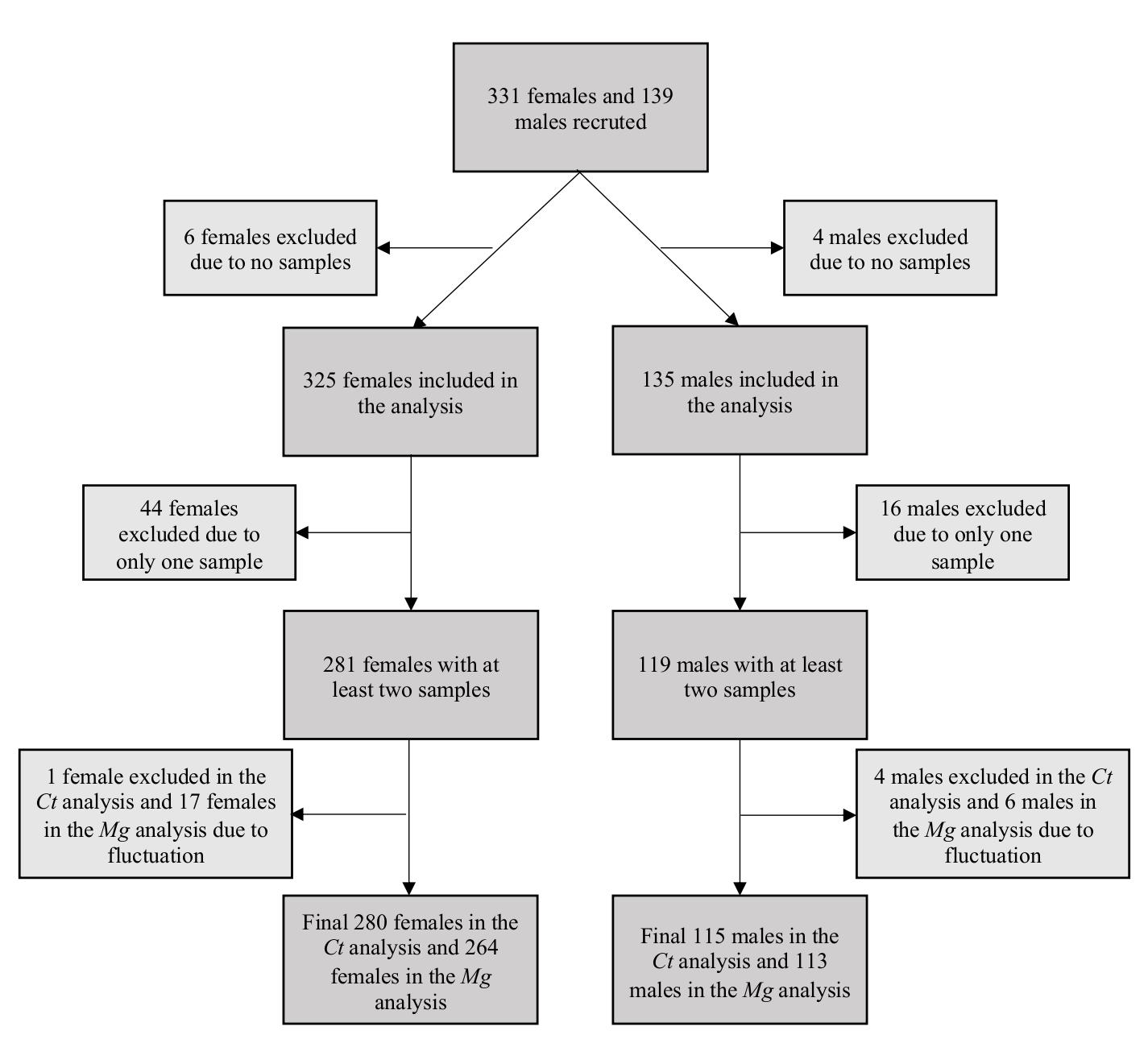

Supplement: Koskela et al. supplementary material [file S095026882500007Xsup001.zip › S095026882500007Xsup001.jpg]
